# Zhangfei/CREB-ZF – A Potential Regulator of the Unfolded Protein Response

**DOI:** 10.1371/journal.pone.0077256

**Published:** 2013-10-14

**Authors:** Rui Zhang, Noreen Rapin, Zhengxin Ying, Erika Shklanka, Timothy W. Bodnarchuk, Valerie M. K. Verge, Vikram Misra

**Affiliations:** 1 Department of Microbiology,Western College of Veterinary Medicine, University of Saskatchewan, Saskatoon, Saskatchewan,Canada; 2 Department of Anatomy and Cell Biology and Cameco Multiple Sclerosis Neuroscience Research Center, University of Saskatchewan, Saskatoon, Saskatchewan, Canada; University of Hong Kong, Hong Kong

## Abstract

Cells respond to perturbations in the microenvironment of the endoplasmic reticulum (ER), and to the overloading of its capacity to process secretory and membrane-associate proteins, by activating the Unfolded Protein Response (UPR). Genes that mediate the UPR are regulated by three basic leucine-zipper (bLZip) motif-containing transcription factors – Xbp1s, ATF4 and ATF6. A failure of the UPR to achieve homeostasis and its continued stimulation leads to apoptosis. Mechanisms must therefore exist to turn off the UPR if it successfully restores normalcy. The bLZip protein Zhangfei/CREBZF/SMILE is known to suppress the ability of several, seemingly structurally unrelated, transcription factors. These targets include Luman/CREB3 and CREBH, ER-resident bLZip proteins known to activate the UPR in some cell types. Here we show that Zhangfei had a suppressive effect on most UPR genes activated by the calcium ionophore thapsigargin. This effect was at least partially due to the interaction of Zhangfei with Xbp1s. The leucine zipper of Zhangfei was required for this interaction, which led to the subsequent proteasomal degradation of Xbp1s. Zhangfei suppressed the ability of Xbp1s to activate transcription from a promoter containing unfolded protein response elements and significantly reduced the ability to Xbp1s to activate the UPR as measured by RNA and protein levels of UPR-related genes. Finally, specific suppression of endogenous Zhangfei in thapsigargin-treated primary rat sensory neurons with siRNA directed to Zhangfei transcripts, led to a significant increase in transcripts and proteins of UPR genes, suggesting a potential role for Zhangfei in modulating the UPR.

## Introduction

One of the main functions of the endoplasmic reticulum (ER) is to modify, process and fold proteins destined for secretion or insertion into membranes. The ER also plays critical roles in maintaining intracellular calcium stores, steroid and lipid biosynthesis, membrane regeneration and gluconeogenesis (reviewed in 1,2). Since protein folding and processing require an oxygen rich microenvironment, as well as adequate nutrient and calcium levels, deficits in these components lead to the accumulation of unfolded or inadequately modified proteins. The ER then initiates a program of recovery called the Unfolded Protein Response (UPR, reviewed in 3,4). The UPR has three main goals: the degradation of misfolded proteins, suppression of the synthesis of new proteins, and an increase in the synthesis of chaperones and other proteins required for processing. The suppression of additional protein synthesis is achieved by the phosphorylation of the eukaryotic translation initiation factor 2a (eIF2α) by the ER-stress sensor - double stranded RNA protein kinase-like ER kinase (PERK [5]). In addition, three basic leucine-zipper motif (bLZip) containing proteins: spliced X-box binding protein (Xbp1s), Activation transcription factor (ATF)4 and ATF6, activate the transcription of UPR-related genes. The protein Xbp1s results from the unique extra-nuclear splicing of the mRNA for the transcriptionally inactive protein Xbp1u by the ER stress sensor, inositol-requiring enzyme/ER to nucleus signaling protein (IRE1/ERN1). Xbp1s retains the basic leucine-zipper motif (bLZip) coded by the unspliced Xbp1u mRNA but acquires a transcription activation domain and a nuclear transport motif [6]. A failure of the UPR to re-establish normalcy triggers apoptosis while successful homeostasis leads to suppression of the UPR.

The UPR includes feed back mechanisms that mediate a retraction of the UPR if ER function is restored. The proteins GADD34 [7,8], Nck1 [9,10] and p58iPK ([11,12] and reviewed by [13] recruit protein phosphatases that dephosphorylate eIF2a restoring protein synthesis. The protein Xbp1u, dimerizes with Xbp1s and ATF6 and targets them for proteasomal degradation [14,15]. With the exception of Xbp1u, most of the UPR-modulating mechanisms described to date are aimed at the PERK effector pathways of the UPR. Relatively little is known about the suppression of the IRE1 and ATF6 arms of the response.

Zhangfei/CREBZF/SMILE was first discovered as a binding partner for Host Cell Factor (HCF), a co-activator of the herpes simplex virion transcription factor VP16 [16]. Translation for the protein is initiated at two alternate initiation codons [17], although both isomers appear to have similar properties. The primary structure of the protein contains a leucine zipper, a basic region that lacks an asparagine residue conserved in most bLZip proteins, three potential nuclear factor binding domains (LLXXLL, where L is a leucine residue and X is any amino acid), and a domain for binding HCF. Zhangfei interacts with several proteins, possibly through its nuclear receptor and HCF binding domains as well as its leucine zipper. While Zhangfei can activate gene expression through factors such as p53 [18] and ATF4 [19], it suppresses the activity of a number of transcription factors which include nuclear receptors [17,20,21], bLZip containing proteins such as CREBH [22] and Luman/CREB3 [23], SMAD 1,5,8 [24] and herpes simplex virion associated VP16 [25]. We have detected Zhangfei protein in differentiated neurons, but not in developing neurons or cells of neuronal tumours [25]. The ectopic expression of Zhangfei in medulloblastomas and other tumours causes the cells to stop growing and eventually to die [26–28]. Zhangfei suppresses the ability of Luman/CREB3 [23] and CREBH [22], to activate transcription. Since these proteins are known to regulate the UPR in some cell types, we hypothesized that Zhangfei may be involved in modulating the UPR. Here we show that Zhangfei can suppress the expression of UPR genes activated in response to the drug thapsigargin. We further show that this effect is mediated, at least partially, by the leucine-zipper dependent interaction of Zhangfei and Xbp1s resulting in the proteasomal degradation of Xbp1s. Our results support the hypothesis that Zhangfei has the capacity to modulate the UPR.

## Materials and Methods

### Cell Culture

The human medulloblastoma cell line ONS-76 [29], was obtained from Michael Taylor (University of Toronto). These cells were cultured as described previously [27]. Vero cells were obtained from the American Type Tissue Culture Collection and grown in Dulbecco’s minimal essential medium containing penicillin and streptomycin and 10% newborn calf serum. All media, serum and antibiotics were purchased from Invitrogen. For some experiments cells were treated with 100 nM thapsigargin or with an equivalent amount of DMSO, the diluent for thapsigargin, for 4 hr. The duration of thapsigargin treatment was based on preliminary experiments to determine the time course of the accumulation of selected UPR-related transcripts following thapsigargin treatment.

### Immunofluorescence

Cells were processed for immunofluorescence as described previously [30]. Cells were stained for Zhangfei (or its mutant) using anti-FLAG monoclonal antibody (Sigma, 082K9164), for other proteins - rabbit anti-GRP78 serum (Abcam, ab21685), for anti-HERP (Abcam, ab73669-100) and anti-Xbp1 (Abcan, ab37152). Secondary antibodies were Alexa Fluor 488 linked anti-mouse and Alexa Fluor 546 linked anti-rabbit antibodies (Invitrogen, A-11001, A-11035). Cell nuclei were stained using Hoechst fluorescent dye (Molecular Probes, 33342).

### Plasmids

The construction of pcZF [16], a plasmid that expresses Zhangfei in mammalian cells, pCAT3BATF6 [23], with the coding sequences for chloramphenicol acetyl transferase (CAT) and pMZF [16], with the coding sequences for Zhangfei linked to the DNA-binding domain of yeast GAL4, have been described. Plasmid pCGNATF6 (1-373), which expresses the constitutively active truncated form of ATF6, and p5XATF6GL3, which contains 5 copies of the UPRE-containing oligonucleotide, CTCGAGACAGGTGC**TGACGTGG**CATTC, were obtained from Ron Prywes, Columbia University, USA [31]. A plasmid expressing the functionally active, spliced form of Xbp1 cDNA was obtained from K. Mori, Kyoto University, Japan [32]. The plasmid, pG5EC, a CAT reporter plasmid with 5 copies of the yeast Gal4-UAS as well as the pM series of plasmids for constructing Gal4 fusion proteins were obtained from I. Sadowski, University of British Columbia, Canada [33]. To construct pcZF(L/A), an expression vector in which the codons for the first six consecutive leucines in the leucine-zipper of Zhangfei were replaced by codons for alanine, a 265 bp synthetic DNA fragment (IDT) bracketed by NotI and SgrA1sites was used to replace a fragment between unique Not1 and SgrA1 sites in the coding sequences of Zhangfei in pcZF. An internal PstI site within the fragment was eliminated with a silent mutation to allow for screening of the mutant. The coding sequences in the mutant were sequenced to confirm the mutation and to ensure that no unintended changes had been made. 

### Adenovirus vectors expressing Zhangfei and β-galactosidase (LacZ)

These vectors were constructed, grown, and purified using the Adeno-X Expression System (Clontech, K1650-1). They were created in our laboratory as described earlier [23]. ONS-76 cells were infected with Adeno-Zhangfei, Adeno-LacZ (expressing *E. coli* β-galactosidase, LacZ) or mock-infected. A multiplicity of infection (MOI) of 100 plaque forming units (PFU) per cell was used. 

### mRNA purification and cDNA synthesis

RNA was purified using RNeasy Plus Mini Kit (Qiagen, 74136) and cDNA synthesized using Quantitect Reverse Transcription Kit (Qiagen, 2053414). One μg of template RNA at a time was converted to cDNA. To ensure high quality RNA for qRT-PCR array analysis, samples were analyzed by electrophoresis on an Agilent 2100 Series Bioanalyzer, Eukaryotic Total RNA Nano series II, version 2.0.

### qRT-PCR arrays and PCR confirmation

Unfolded protein response qRT-PCR arrays were purchased (SABiosciences, array number PAHS-089A-12). The array contained primers for 84 gene transcripts involved in the UPR. The array also contained controls for genomic DNA contamination and reverse transcriptase efficiency. The results from triplicate experiments were analyzed by using a SABiosciences online resource called RT^2^ profiler. To confirm the results of the qRT-PCR array we designed primers (Table S1) for the activated or repressed genes using human mRNA gene sequences found on the NCBI human genome website (http://www.ncbi.nlm.nih.gov/projects/genome/guide/human/). Sequences were designed using the website tool Primer3 (http://frodo.wi.mit.edu/primer3/) and purchased from Integrated DNA Technologies (IDT). For the normalizer, GAPDH, primers have been described ^25^. Agilent Technologies’ Brilliant II SYBR Green QPCR Master Mix Kit (catalog number 600828) was used to perform qRT-PCR (in triplicate, also repeated 3 times). The PCR machine used was a Stratagene Mx3005P model. Cycle details are as follows: 95°C for 10min, followed by 40 cycles (95°C for 30 sec, 55°C for 1 min, 72°C for 1 min) and a final step (95°C for 1 min, 55°C for 30 sec, 95°C for 30 sec).

Oligonucleotide primers used and their nucleotide sequences are listed in Table S1. All qRT-PCR reactions satisfied MIQE guidelines [34]: Disassociation profiles in reactions that yielded products contained single homogeneous peaks. In all reactions GAPDH was used as a normalizer. In previous qRT-PCR arrays comparing Zhangfei expressing and non-expressing cells five house keeping genes were analyzed. The levels of GAPDH were not affected by Zhangfei expression.

### Co-immunoprecipitation

Vero cells in 6-well dishes were transfected using Lipofectamine 2000 (Invitrogen, 11668-019) with plasmids expressing Xbp1s alone or in combination with a plasmid expressing Zhangfei, tagged at its amino terminus with a FLAG epitope. Twenty-four hr after transfection MG132 (5μM) was added. After an additional 24hr cells were washed with PBS and lysed in 250μl/well cold lysis buffer (50mM Tris, pH7.5, 150mM NaCl, 1mM EDTA and 0.1% TritonX-100) containing protease inhibitor cocktail (Sigma, P8340). After centrifugation at 13,000 xg at 4°C mouse anti-FLAG antibody (5μl) was added to the supernatant and the sample incubated for 12hr with constant gentle agitation. Protein A/G agarose beads (100μl, Fisher Scientific, 20421) was added and the samples were incubated for an additional 4hr at 4°C. Agarose beads were collected by centrifugation at 13,000 xg and washed 4 times in lysis buffer before boiling in SDS-PAGE sample buffer. Proteins in samples of the unfractionated cell lysate or immunoprecipitates were separated by SDS-PAGE, transferred to membranes and probed with either rabbit anti-Xbp1 (Abcam, ab37152) or anti-ZF antisera. Antibodies were visualized after incubation with Alexa488-labelled anti-rabbit antibody (Invitrogen, A-11001).

### Adult DRG culture

The study was carried out in strict accordance with The Canadian Council on Animal Care and the University of Saskatchewan animal care committee guidelines. The protocol (Role of neurotropic molecules in intact and injured neurons, protocol #19920164) was approved by the University of Saskatchewan Committee on Animals Care (UCACS) and the Animal Research Ethics Board (AREB). Dorsal root ganglia (DRG) were removed from adult male Wistar rats, treated with 0.25% collagenase (Sigma, C-0130) for 1 h at 37°C and then dissociated with 2.5 % trypsin (Sigma) for 30 min at 37°C before being plated on laminin (1 g/mL, BD Biosciences, 354232 ) and poly-D-lysine-coated (25 g/mL, Sigma) coverslips at 10^4^ cells per well in a 6-well plate (BD biosciences) in DMEM (Sigma Life Science) supplemented with 10ng/ml of NGF (Cedarlane, CLMNET-005.1). Cytosine ß-D-arabinofuranoside (Ara-C, 10 M; Sigma, C-6880) was included to inhibit proliferation of non-neuronal cells. Rat neurons were transfected using Lipofectamine RNAiMAX (Invitrogen, 13778-150) with plasmids expressing either siRNA against Zhangfei or control siRNA. In previous experiments we had shown that this siRNA reduces Zhangfei protein levels by almost 50% and abrogates the ability of the protein to suppress the transcription factor Luman/CREB3 [35]. Twenty four hr after transfection, cells were treated with 100nM thapsigargin (Sigma, T-9033) for 4 hr. Cells were then harvested, RNA purified and UPR-related transcripts as well as ZF transcripts assessed by qRT-PCR as described above. To assess the efficiency with which primary neurons could be transfected with the procedure used, DRG neurons were transfected at a final concentration of 10nM with the TYE 563 DS Transfection Control duplex (Integrated DNA Technologies) using Lipofectamine RNAiMAX Reagent (Life Technologies) according to the manufacturer's instructions. Cells were imaged 24 hours post transfection.

## Results

### Does the ectopic expression of Zhangfei influence the UPR?

**Figure 1:**
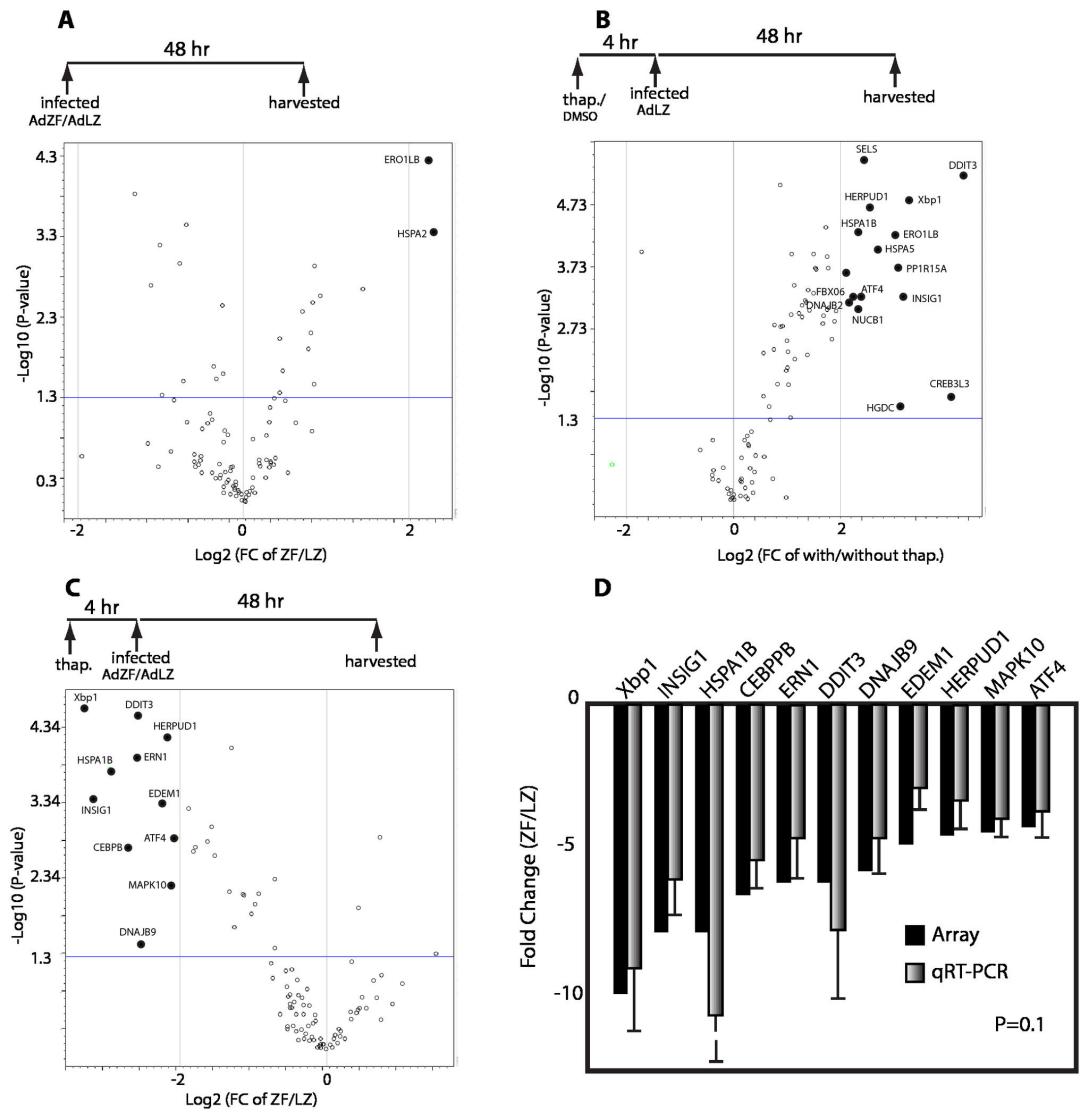
Suppression of UPR genes by Zhangfei in ONS-76 medulloblastoma cells treated with thapsigargin. *A, B, C* – “Volcano” plots from data derived from qRT-PCR arrays designed to monitor transcripts of genes associated with the UPR. The horizontal axes in these graphs represent log_2_ fold differences between the samples indicated. The vertical axis represents -log_10_ of P values for data derived from three pairs of arrays. The horizontal line (-1.3) represents a P value of 0.05. Transcripts that changed more than 2 fold (<-2 or >2, vertical lines) and had a P value <0.05 were considered significant and are indicated by solid spots with gene designations. A. Difference in UPR transcripts between ONS-76 cells expressing either Zhangfei or LacZ. Cells were infected with adenoviruses expressing either Zhangfei or LacZ and harvested 48 hr later for RNA extraction and analysis. B. Differences between LacZ-expressing cells treated with either thapsigargin or DMSO (solvent for thapsigargin). Cells were treated for 4 hr then infected with adenovirus vector expressing LacZ and harvested for RNA extraction and analysis 48 hr later. C. Effect of Zhangfei on UPR genes activated by thapsigargin. Cells were treated with DMSO or thapsigargin for 4 hr then infected with adenovirus vectors expressing either Zhangfei or LacZ and harvested 48 hr later for RNA extraction and analysis. D. A comparison of data from the qRT-PCR arrays (C.) with data from qRT-PCR experiments using primers designed “in-house”. ATF4 – activation transcription factor 4CEBPB - CCAAT enhancer binding protein-beta, DDIT3 – DNA damage inducible transcript -3, DNAJB9 – homologue of DNAJ/ 40 kD heat shock protein, EDEM – ER degradation enhancer mannosidase alpha-like 1, ERN1 – ER to nucleus signaling, HERPUD1 – homocysteine-inducible ER stress inducible ubiquiti-like domin member 1, HSPA1B – heat shock 70 kD protein 1B, INSIG1 – insulin-induced gene 1, MAPK10 – mitogen-activated protein kinase 10, Xbp1 - X box binding protein 1.

We used qRT-PCR arrays designed to assess genes related to the UPR to determine if the expression of Zhangfei in cells influenced the response. Since we have shown that Zhangfei has a profound influence when ectopically expressed in ONS-76 human medulloblastoma cells [27,35], we compared RNA purified from ONS-76 cells infected with adenoviruses expressing either Zhangfei (AdZF) or the control protein β-galactosidase (AdLZ, Figure 1A). Except for two transcripts that were present in a two–fold excess in Zhangfei-expressing cells, we found no significant differences in the two samples. Since cells in the laboratory normally grow in unstressed conditions, we then determined if the calcium ionophore thapsigargin would induce UPR genes. Cells were treated with 100nM thapsigargin or with an equivalent amount of DMSO, the diluent for thapsigargin, for 4hr. The drug was then removed and cells infected with AdLZ. Forty eight hr later RNA was purified, converted to cDNA and analyzed with UPR qRT-PCR arrays (Figure 1B). In thapsigargin-treated cells transcripts for several UPR-related genes were significantly increased. We next compared thapsigargin-treated cells infected with either AdZF or AdLZ. Figure 1C shows that many UPR-related gene transcripts were reduced in Zhangfei-expressing cells. To confirm these results we independently designed primers to amplify portions of the mRNAs for the genes identified by the arrays. Results using these primers confirmed the results of the arrays (Figure 1D). 

**Figure 2:**
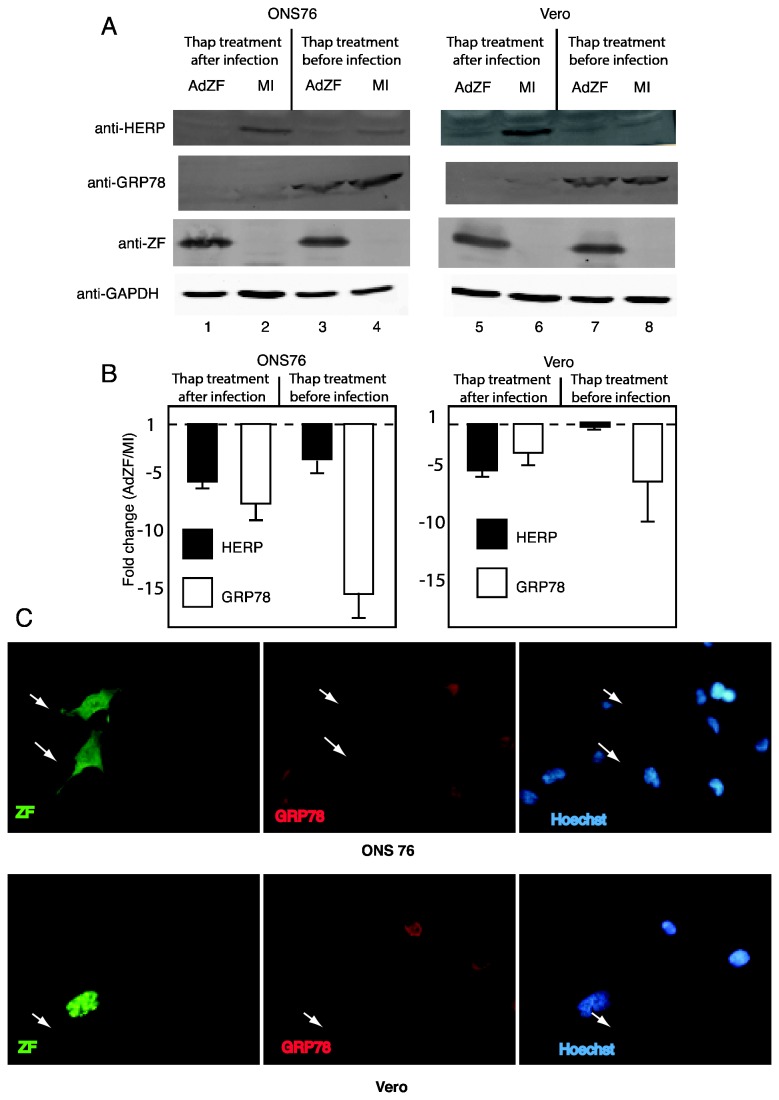
Effect of Zhangfei on HERP and GRP78 transcripts and proteins in cells treated with thapsigargin. ONS76 and Vero cells were treated for 4 hr with thapsigargin either after infection, or mock infection, with an adenovirus vector expressing ZF (lanes 1,2, 5 and 6) or before infection (lanes 3,4, 7 and 8). Cells were harvested 24 hr after infection. Proteins in samples were separated by SDS-PAGE and probed with antibodies against HERP, GRP78 or Zhangfei (ZF) and GAPDH (A) and RNA was extracted from parallel duplicate cultures and assayed for HERP and GRP78 transcripts by qRT-PCR (B). C. ONS-76 and Vero cells were transfected with plasmids expressing FLAG epitope linked to the coding sequences of Zhangfei. Cells were fixed and incubated with a mixture of mouse monoclonal antibody against FLAG and rabbit antibody against GRP78 followed by Alexa 488-linked anti-mouse and Alexa 546-linked anti-rabbit antibodies. Cells were also stained with Hoechst to stain nuclei. Arrows identify Zhangfei (ZF) expressing cells.

To determine whether a decrease in the transcripts for UPR genes was reflected in a decrease in proteins as well, we simultaneously detected in both ONS76 and Vero cells, protein (Figure 2A) and RNA (Figure 2B) for HERP and GRP78, two components of the UPR. Cells were mock-infected (MI) or infected with an adenovirus vector expressing Zhangfei. We examined cells that expressed Zhangfei for 24 hr and then treated with thapsigargin for 4 hours (lanes 1, 2, 5 and 6). This would determine the ability of cells expressing Zhangfei to respond to the UPR. As an alternative, we treated cells with thapsigargin for 4hr and then infected or mock infected cells (lanes 3, 4, 7 and 8). This would assess the ability of Zhangfei to turn off the UPR once it had been activated. In both experiments, mock-infected cells were an indication of the level of HERP and GRP78 without Zhangfei at the time of cell harvest. Cells already expressing Zhangfei (compare lane 1 with 2, and 5 with 6 in Figure 2A) were unable to make detectable amounts of HERP as compared to mock-infected cells. After 4 hr of thapsigargin treatment mock-infected cells made relatively little GRP78 (barely visible as a faint band in lanes 2 and 6) and even this was not detected in Zhangfei-expressing cells (lanes 1 and 5). When cells were treated with thapsigargin before infection or mock-infection, and then maintained in thapsigargin-free medium for 24 hr, substantial amounts of GRP78 was detected in mock-infected cells (lanes 4 and 8) and this was considerably reduced in Zhangfei–expressing cells (lanes 3 and 7 – the decrease for Vero cells was less than for ONS76 cells). Zhangfei also reduced HERP as compared to mock-infected cells (compare faint band in lane 4 with lane 3). Figure 2B shows that in this experiment Zhangfei, before or after thapsigargin treatment, reduced levels of HERP and GRP78 transcripts. To confirm the effects of Zhangfei on GRP78 we transfected ONS76 and Vero cells with a plasmid expressing Zhangfei and then visualized ZF and GRP78 by immunofluorescence (Figure 2C). Both cell lines cells expressing Zhangfei lacked detectable GRP78.

### Can Zhangfei suppress the ability of Xbp1s to activate transcription and is its leucine-zipper required?

**Figure 3:**
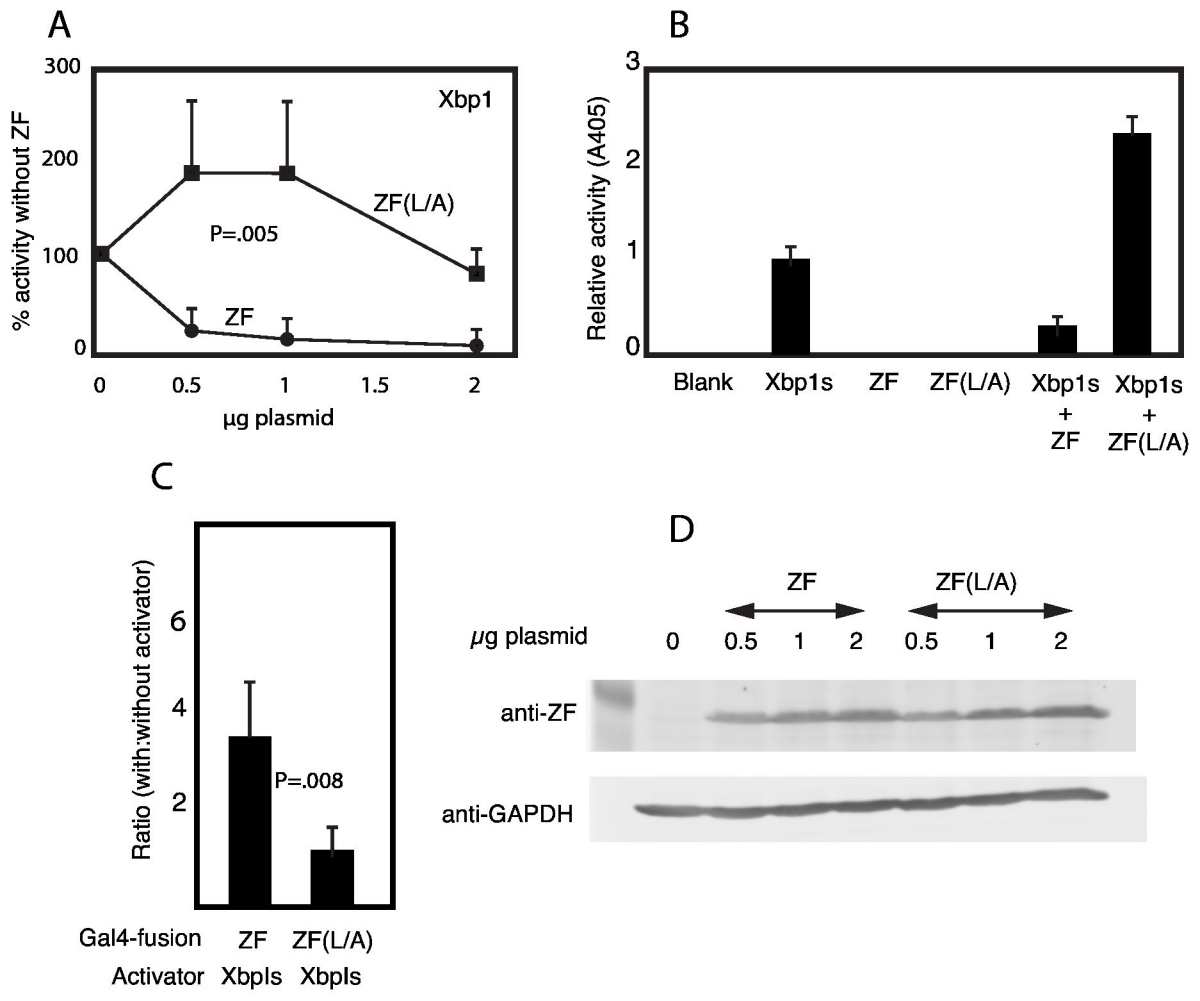
*Zhangfei suppresses* the ability of Xbp1s to activate transcription and requires its leucine zipper to do so. A and B. Vero cells were transfected with a plasmid containing the coding sequence for CAT linked to a promoter with three copies of the unfolded protein response element as well as a plasmid expressing Xbp1s and varying amounts of plasmids expressing either Zhangfei (ZF) or a mutant, ZF(L/A) in which all leucine residues in the LZip domain had been replaced with alanines. All samples also contained, as a control, a plasmid expressing β-galactosidase. The CAT activity in each sample was normalized to this internal control and expressed as a percentage of the activity in samples containing no vector expressing either ZF or ZF (L/A). The total amount of DNA in each transfection was made up to 5μg with “empty” expression vector (pcDNA3). Bars indicate standard deviation from the mean. B. ZF(L/A) does not activate a promoter containing UPRE but enhances the activity of Xbp1s. C. ZF interacts with Xbp1 with its leucine zipper. Cells were transfected with a vector with the coding sequence for CAT linked to three copies of a sequence, UAS, that binds the DNA-binding domain of the yeast protein GAL4. Cells also received plasmids expressing either ZF or ZF(L/A) linked to the Gal4 DNA-binding domain and either an “empty” expression vector or vectors expressing Xbp1s. Bars represent the ratio of the relative CAT activity (normalized to the internal control, β-galactosidase) of samples with Xbp1s to samples with no activator (“empty” vector). D. An immunoblot showing that vectors with cloned ZF or ZF (L/A) express the proteins in a dose-dependent manner. The results represent the averages of three experiments assayed in duplicate. Bars in all figures represent standard deviation from the mean and p values are indicated on the figures.

Since the published literature suggests that at least in some cells Zhangfei is relatively ineffective against ATF6 [22], we examined the effect of Zhangfei on the UPR-regulating transcription factor Xbp1s. In transiently transfected cells Xbp1s can activate transcription of reporter genes linked to unfolded protein response elements (UPRE). To determine if Zhangfei could suppress this ability of Xbp1s, Vero cells were transfected with a CAT-reporter plasmid linked to multiple copies of UPRE (Figure 3 A and B). Cells were also transfected with plasmids expressing the spliced form of Xbp1, Xbp1s, and increasing amounts of a plasmid expressing Zhangfei (Figure 3A). Zhangfei inhibited the activity of Xbp1s in a dose-dependent manner (solid circles). The immunoblot in Figure 3D shows that increasing the amount of the Zhangfei-expressing plasmid in the transfection mixtures also led to increased amounts of the protein in the cells. To determine if the leucine-zipper of Zhangfei was required for its suppressive activity, parallel cultures in the experiments described above were transfected with a plasmid expressing a mutant of Zhangfei in which the first 6 consecutive leucine residues in the bLZip domain were replaced with alanine residues (ZF(L/A)). Unlike Zhangfei, ZF(L/A) did not suppress Xbp1s (Figure 3, A,B). Figure 3D shows that the mutations in ZF(L/A) did not affect its stability. Interestingly, not only did ZF(L/A) not suppress Xbp1s, it consistently appeared to enhance the activities of Xbp1s almost 2 fold (Figure 3A and B). This enhancement did not reflect an increased ability of ZF(L/A) to activate transcription as neither Zhangfei nor ZF(L/A) on their own, had any effect on a UPRE containing promoter (Figure 3B).

To confirm that Zhangfei required its leucine zipper to interact with Xbp1s, we conducted two-hybrid assays in which activation of a reporter gene depended on interaction of Xbp1s with Zhangfei or ZF(L/A) tethered to a promoter by the DNA-binding domain of the yeast transcription factor Gal4 (UAS). Cells were transfected with a reporter plasmid with a Gal4 UAS containing promoter and either Gal-ZF or Gal-ZF(L/A) with or without plasmids expressing Xbp1s. The results in Figure 3C show that the reporter activity in the presence of Gal-ZF was significantly greater than the activity in the presence of Gal-ZF(L/A).

### How does Zhangfei suppress Xbp1?

**Figure 4:**
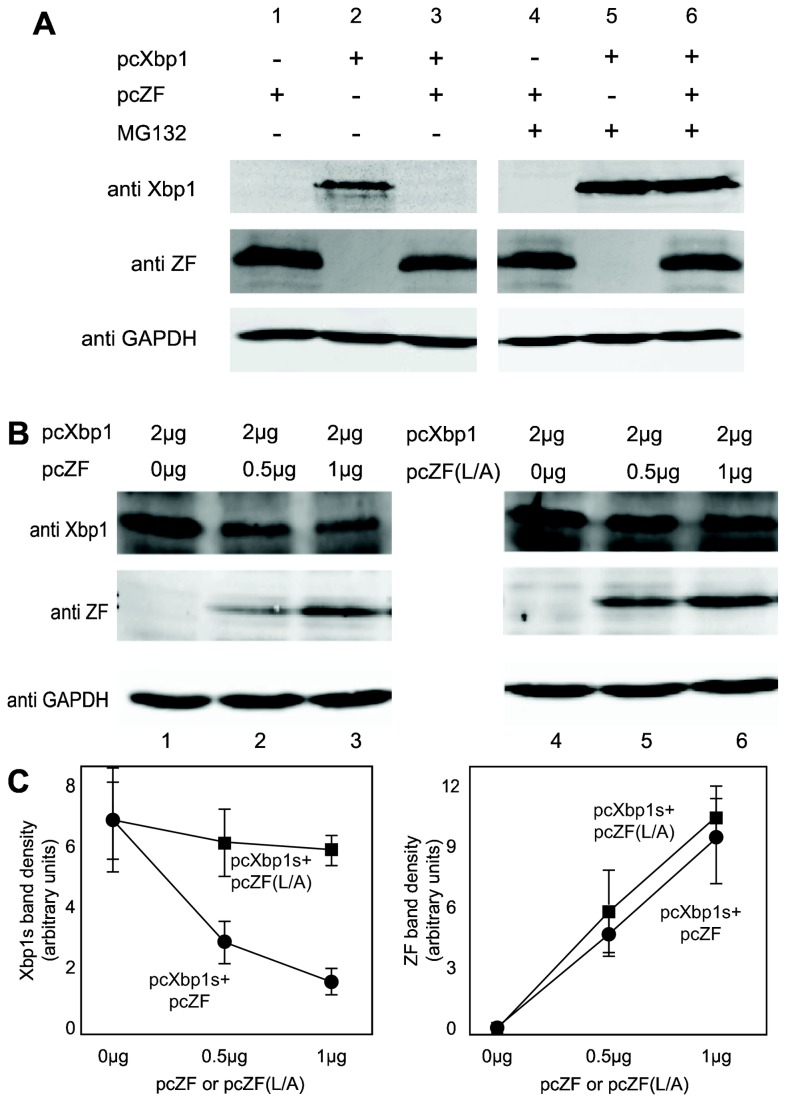
*Zhangfei directs* Xbp1 for proteasomal degradation and requires its leucine zipper to do so. A. Vero cells were transfected as indicated with plasmids expressing Xbp1s and an empty vector or plasmid expressing Zhangfei. Cells either received 5μM MG132 or an equivalent volume of carrier, DMSO. Cells were harvested 24 hr later and proteins detected by immunoblotting. B. Vero cells were transfected with a plasmid expressing Xbp1s alone or increasing amounts of a plasmid expressing Zhangfei or a mutant in which all leucine residues in the zipper had been changed to alanine - pcZF(L/A). Cells were harvested 24 hr later and proteins detected by immunoblotting. The density of each band on the immunoblot was estimated by densitometry and normalized to the density of the GAPDH band in the sample. Average values and standard deviation from three experiments are shown in (C).

**Figure 5:**
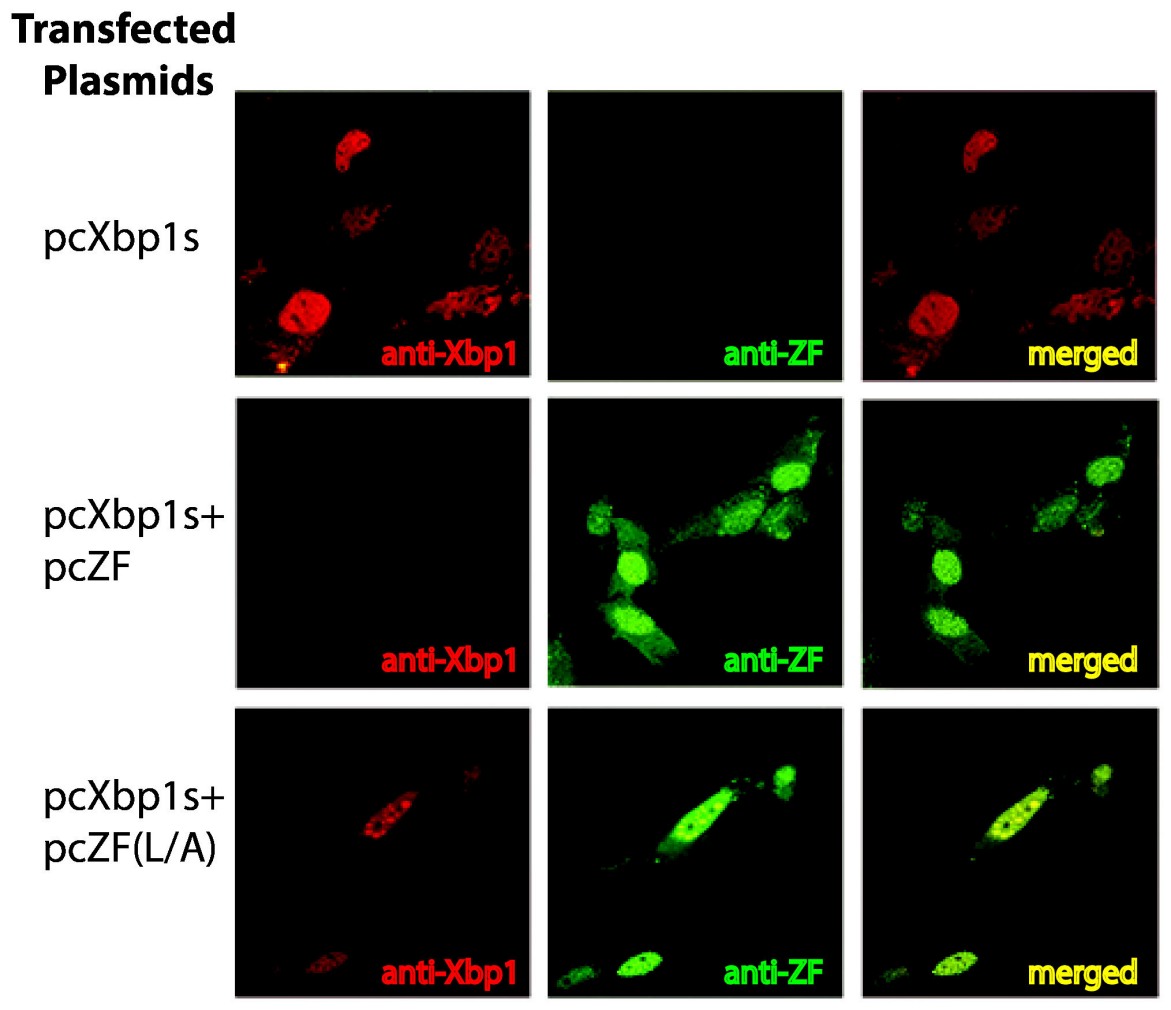
Immunofluorescent images showing the absence of Xbp1 in cells expressing Zhangfei but not *Zhangfei with* a mutated leucine zipper. Vero cells were transfected with plasmids expressing either Xbp1 alone or in combination with either Zhangfei or ZF(L/A). Cells were then fixed and incubated with a mixture of rabbit anti-Zhangfei and mouse anti-Xbp1 antibodies. Antibodies were visualized by staining with Alexa546 (red) anti mouse and Alexa488 (green) anti-rabbit antibodies.

Zhangfei may suppress Xbp1s by either causing its degradation, inhibiting its ability to bind to cognate promoters or by sequestering it from the nucleus. To test the first possibility we examined Xbp1s in cells expressing Zhangfei. Vero cells were transfected with either a plasmid expressing Xbp1s alone or with a plasmid expressing Zhangfei. In cells expressing both proteins we were unable to detect Xbp1s (Figure 4A, compare lane 2 with lane 3). In contrast, the co-expression of both proteins did not lead to an appreciable decrease in Zhangfei (compare lanes 1 and 3). The proteasome inhibitor, MG132 restored Xbp1s levels in Zhangfei-expressing cells (lane 6). To determine whether the leucine zipper of Zhangfei was required for the induced degradation of Xbp1s, we expressed Xbp1s with increasing amounts of plasmid expressing either Zhangfei or ZF(L/A). Figure 4B (Figure 4C shows densitometer measurements of repeated experiments) shows that at low concentrations protein (0.5 and 1μg expression plasmid) there was an obvious difference in the ability of Zhangfei and its LZip mutant to induce the degradation of Xbp1s. At higher concentrations (2μg of plasmid, not shown) the differences between the mutant and wild-type Zhangfei were less pronounced. Figure 5, which shows intracellular proteins detected by immunofluorescence, supports these data – we were unable to detect Xbp1s in cells expressing Zhangfei, while the LZip mutant, ZF(L/A), had no effect. When Xbp1s and ZF(L/A) were expressed together both proteins colocalize in the nucleus (Figure 5, bottom row) with higher concentrations in nuclear structures. This suggests that nuclear localization of Xbp1s does not rely on association with Zhangfei.

### Does Zhangfei interact with Xbp1s?

**Figure 6:**
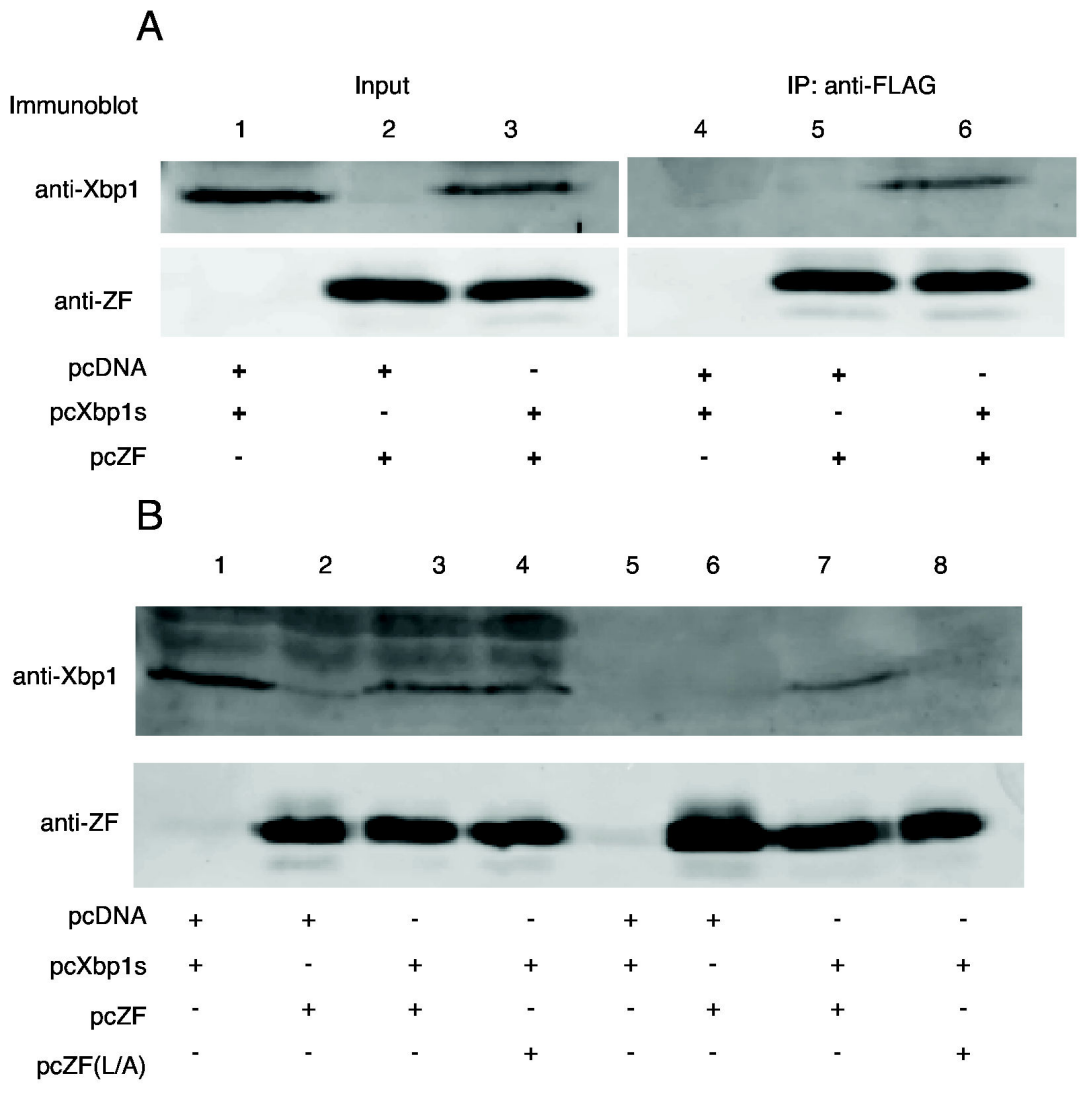
Zhangfei co-immunoprecipitates with Xbp1s in MG132-treated cells. A. Vero cells were transfected to express Xbp1s and FLAG-tagged Zhangfei either on their own alone or together. The cells were treated with MG132 to reduce proteasomal degradation and, 24 hr later, cells lysates were immunoprecipitated with mouse anti-FLAG antibody. The precipitates were separated by SDS-PAGE and Xbp1 and Zhangfei detected in immunoblots with rabbit anti-Xbp1 or anti-Zhangfei antisera. Lanes 1-3, represent cell lysates without immunoprecipitation while lanes 4-6 are immunoblots of material precipitated by anti-FLAG antibodies. B. A similar experiment as in A, showing that Xbp1s was not co-precipitated with the Zhangfei mutant ZF(L/A).

To determine if Zhangfei and Xbp1s interacted, we co-expressed the proteins in Vero cells. Zhangfei coding sequences included a FLAG epitope. Since our previous experiments indicated that interactions between the two proteins might lead to the proteasomal degradation of Xbp1s (Figure 4A), we treated cells with MG132 to suppress degradation. From the lysates of these cells we precipitated Zhangfei and associated proteins with monoclonal antibodies against FLAG and then detected Xbp1 or Zhangfei in the immunoprecipitates using immunoblots antisera against either Xbp1 or Zhangfei. Figure 6A shows that in cells expressing both proteins they were in a stable association (lane 6). In a similar experiment, Xbp1 did not precipitate with ZF(L/A) (Figure 6B, compare lanes 7 and 8) confirming our results that the leucine-zipper of Zhangfei was required for the interaction.

###  Can endogenous Zhangfei suppress the UPR in sensory neurons?

**Figure 7:**
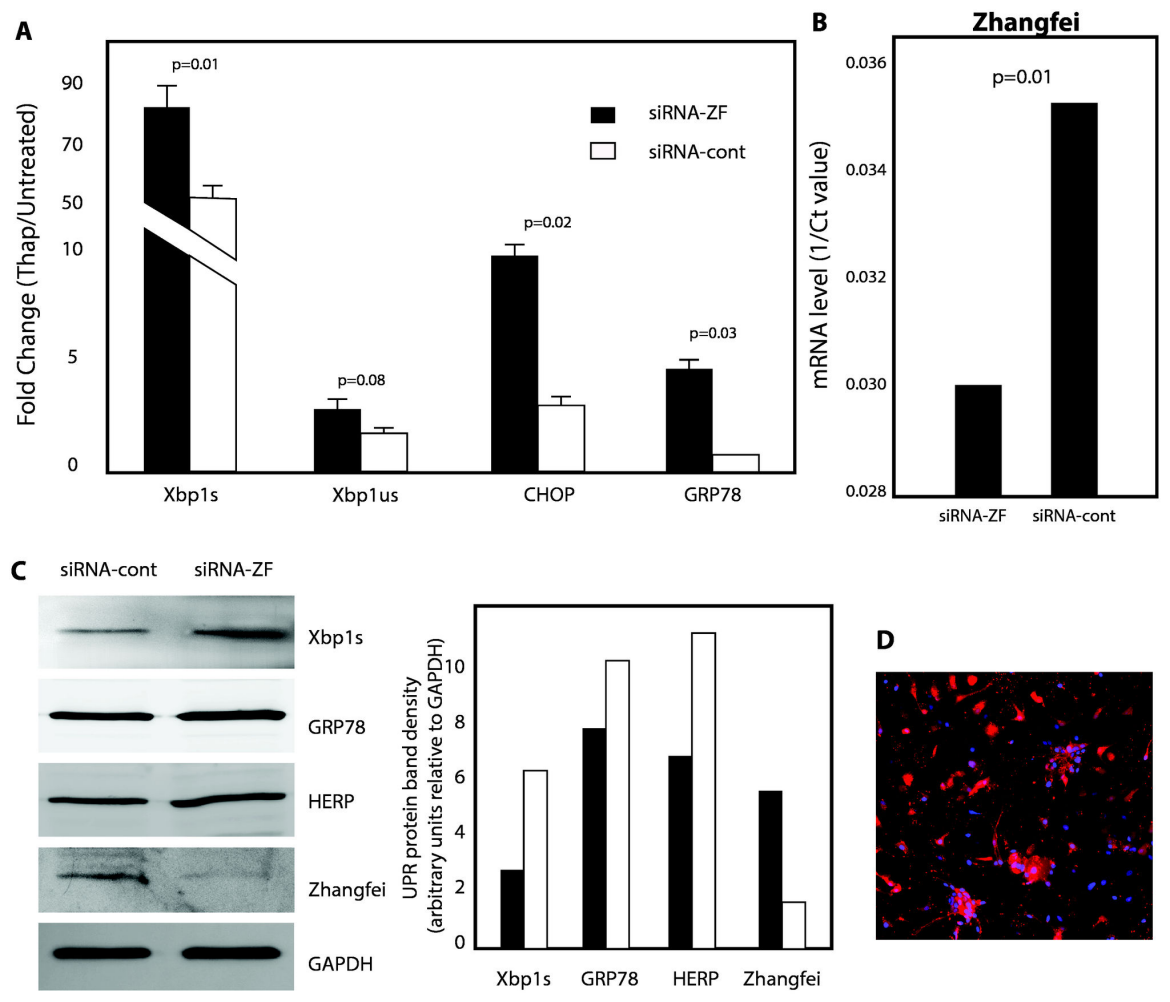
Endogenous *Zhangfei suppresses* the activation of UPR genes in rat peripheral neurons. Primary rat dorsal root neurons were transfected with plasmids expressing either control siRNA or siRNA against Zhangfei (siRNA-ZF). The next day cells were treated with DMSO or thapsigargin and 4hr later RNA was harvested for qRT-PCR analysis using primers designed to amplify Xbp1s, CHOP, GRP78, Xbp1us (A) or Zhangfei (B). A. Fold increases in RNA between DMSO and thapsigargin treated samples comparing siRNA-ZF (black bars) and siRNA control (white bars) expressing cells. B. Effect of si-ZF on transcripts levels of endogenous Zhangfei. Results are expressed as 1/Ct. Columns represent averages of triplicate samples with bars as standard deviation from the mean. C. Suppression of endogenous Zhangfei increases Xbp1s, Grp78 and HERP proteins. Lysates of neurons transfected with either siRNA-ZF or siRNA-control were analyzed by immunoblotting using antibodies against UPR-related proteins. Bands on immunoblots of the left were scanned and band densities relative to the internal standard GAPDH are on the graph on the right. D. Transfection efficiency test of siRNA. Primary rat dorsal root neurons were transfected at a final concentration of 10 nM with the TYE 563 DS Transfection Control duplex, and were imaged 24 hours post transfection (Red: marked siRNA; blue: nuclear).

We had previously detected Zhangfei in mature neurons of the central nervous system and in sensory neurons in trigeminal ganglia [25]. To determine if endogenous Zhangfei could suppress the UPR in these cells, disassociated neurons from adult rat dorsal root ganglia were transfected with plasmids expressing either siRNA against Zhangfei or control siRNA [35]. The UPR was then induced in these cells with thapsigargin. Levels of transcripts for Xbp1s, unspliced Xbp1 (Xbp1us), CHOP, GRP78 and Zhangfei as well as protein levels for Xbp1s, GRP78, HERP, GAPDH and Zhangfei were then measured using qRT-PCR, and immunoblotting and densitometry. The primers used for qRT-PCR were directed against conserved regions of the coding sequences of these genes. The results (Figure 7A and 7B) show that in cells in which siRNA against Zhangfei had reduced its endogenous transcripts and protein (Figure 7B and 7C), transcripts and proteins for several UPR-related genes were increased. There was a trend towards an increase in Xbp1us transcripts although the difference from control siRNA-expressing cells was not significant. These results suggested that endogenous Zhangfei has the capacity to modulate the UPR. To demonstrate that most cells in the primary sensory neuron culture could be transfected we transfected the cells with fluorescent double stranded RNA. Figure 7D shows that most cells in the culture were capable of taking up transfected DNA.

## Discussion

The inability of the UPR to restore normal ER function results in apoptosis. However, a successful homeostatic response should result in modulation of the UPR and resumption of normal cellular function. To do this several UPR induced proteins such as p58IPK [13], NCK1 [9,10] and GADD34 [7,8] feed-back to relieve the inhibition of protein synthesis mediated by PERK. These act by recruiting phosphatases that dephosphorylate eIF2α. In addition, the product of unspliced Xbp1 mRNA binds Xbp1s and ATF6 through bLZip interactions and targets the proteins for proteasomal destruction [14,15].

Our qRT-PCR array data revealed a global significant decrease in transcripts for genes activated by the UPR inducer thapsigargin in ONS-76 human medulloblastoma cells when Zhangfei was expressed in these cells (Figure 1). In contrast, Zhangfei had no effect on the UPR gene transcripts in cells growing normally under favourable non-stressful conditions. This suggests that Zhangfei did not globally suppress transcription in cells but directly or indirectly targeted the expression of certain genes. Mature neurons are one of the few cell types in which we have been able to detect endogenous Zhangfei [25]. In support of our data, we also showed that selective suppression of Zhangfei by siRNA in adult primary sensory neurons from rat dorsal root ganglia increased levels of transcripts and proteins for UPR-related genes (Figure 7) confirming the ability of the protein to suppress the UPR once it had been activated, by thapsigargin in this case.

To determine how Zhangfei might modulate the UPR, we focused on the ability of Zhangfei to suppress the ability of Xbp1s, one of the main bLZip mediators of the UPR. We found that in cells transfected to transiently express Xbp1s as well as Zhangfei, the ability of Xbp1s to activate transcription was suppressed (Figure 3). This was confirmed by a corresponding decrease in the level of the corresponding UPR proteins (Figure 2). While this can be explained, at least to some extent, by the effect of Zhangfei on the ability of Xbp1s to initiate transcription of UPR genes, we are puzzled how Zhangfei suppressed levels of the spliced Xbp1 transcripts themselves. The bLZip transcription factor responsible for Xbp1 transcription has not been unambiguously identified however our earlier data [26] as well as that of others show that activation of the UPR leads to an increase in spliced Xbp1 mRNA without a corresponding increase in unspliced transcripts. In addition, Figure 4 shows that while suppression of Zhangfei by siRNA decreased levels of spliced Xbp1 mRNA it did not have a significant effect on unspliced transcripts. This suggests that Zhangfei may have an effect on the IRE1-mediated cytoplasmic splicing process in addition to suppressing transcription by Xbp1s. 

Basic leucine-zipper motif containing proteins are known to interact with other bLZip proteins through their leucine zippers and can be categorized according to whether they strongly favour the formation of homo-dimers, hetero-dimers or both [36]. In addition, Newman and Keating [37] using peptide arrays and fluorescent bLZip probes, measured interactions between the bLZip regions of all human and yeast bLZip proteins. According to their results the Xbp1 and ATF6 domains were the only ones among the 49 bLZip proteins tested that showed a strong association with the Zhangfei domain. Binding between Zhangfei and ATF4 could only be detected when the fluorescent Zhangfei peptide was used to detect binding to the array.

Since bLZip proteins can act as co-activators by interacting with other transcription factors by mechanisms that do not rely on their bLZip domains [38], we determined whether the suppressive effect of Zhangfei on Xbp1s required its leucine zipper. Our data strongly suggest that it does (Figures 3, 4, 5 and 6). A mutant of Zhangfei in which all 6 consecutive leucines in the zipper were replaced with alanine was less efficient at suppressing Xbp1s than Zhangfei with an intact zipper. Further, our results in the *in vivo* protein hybrid assay in which transcriptional activation relied upon interaction of Gal4-linked Zhangfei or its mutant with the Xbp1s (Figure 3C), as well as our inability to co-precipitate Zhangfei (L/A) and Xbp1s, support our observations.

Interestingly, not only did Zhangfei (L/A) not suppress Xbp1s, it consistently enhanced its ability to activate transcription (Figure 3 A and B). A possible explanation for this phenomenon may lie in the co-localization of the two proteins in nuclear domains (Figure 5, bottom row). We have observed that if proteasomal degradation is suppressed, Zhangfei co-localizes with Luman/CREB3 (another bZip protein that it suppresses) in intranuclear promyelocytic leukemia protein-containing nuclear domains [23]. These domains are sites for nuclear proteasomes [39] and it is possible that, on their own, both Zhangfei and Xbp1s are normally targeted to these sites. Expressed together, Zhangfei enhances the degradation of Xbp1s while Zhangfei (L/A), unable to bind Xbp1s, has a suppressive effect on proteasomes.

The interaction between Zhangfei and Xbp1s results in the proteasomal degradation of Xbp1s (Figure 4). Elucidating the mechanism by which Zhangfei targets Xbp1s for proteasomal degradation will require additional work. However, several bLZip proteins have been shown to target other proteins for such destruction. Thus, the human T-cell leukemia virus coded bLZip protein HBZ and the host transcription factor maculoaponeurotic fibroma (Maf) homologue B interact through their bLZip domains following which MafB is targeted for proteasomal degradation [40]. HBZ also interacts with host interferon response factor 1 (IRF1) and targets it for degradation [41]. Some interactions between bLZip proteins, such as between ATF5 and nucleoplasmin [42] lead to the ubiquitination of the target prior to degradation suggesting a mechanism for proteasomal targeting. Recently a SUMO-conjugase, UBC9, was shown to stabilize Xbp1s by interacting with its bLZip domain. Displacement of UBC9 from Xbp1s by Xbp1u led to decrease in the stability of Xbp1s [42]. Zhangfei may, in a similar manner, destabilize Xbp1s by displacing UBC9 from its bLZip domain. SUMOylation of proteins prevents ubiquitination [43] and subsequent proteasomal degradation. Interestingly, UBC9 did not require its SUMOylating activity to stabilize Xbp1s [42] suggesting that UBC9 exerts its effect by other means. 

While we have shown a direct effect of Zhangfei on Xbp1s, Zhangfei may suppress the other UPR-inducing bLZip factors as well. Although ATF6 is one of the best-characterized of these factors, other ER-resident bLZip proteins such as Luman/CREB3 and CREBH are thought to perform this role in some cell types (reviewed in 44,45). We have previously shown that Zhangfei can suppress the activity of Luman/CREB3 but not ATF6. Recently, Misra and others [22] showed that in hepatoma cells Zhangfei/SMILE suppresses the ability of CREBH to induce UPR genes. While further studies about the role of Zhangfei in regulation the UPR are clearly needed, our results and those of others show that the Zhangfei has the ability to suppress Xbp1 as well as other bLZip proteins that may substitute for the ATF6-arm of the ER-stress sensing pathways. However, since we have only been able to detect Zhangfei in mature, differentiated neurons, its influence is likely restricted to a few cell types. Alternatively, given its dramatic effect on cell division [26,27], it may be expressed in a wider array of cells but only in a very transient manner, when it is needed.

## Supporting Information

Table S1 **Oligonucleotides primers used for Real Time PCR.**
(PDF)
