# Supplementary material for: Zhangfei/CREB-ZF – A Potential Regulator of the Unfolded Protein Response
Source: PLoS One. 2013 Oct 14;8(10):e77256. doi: 10.1371/journal.pone.0077256 (PMC3796484; doi:10.1371/journal.pone.0077256)
Supplement: Table S1 — Oligonucleotides primers used for Real Time PCR. (PDF) [file pone.0077256.s001.pdf]

Gene Name	Symbol	Primer	Derived from (	Sequence	Prod. size (bp)
Activating transcription factor 4	ATF4	ATF4 F	NM_001675	TCA AAC CTC ATG GGT TCT CC	226
		ATF4 R		GTG TCA TCC AAC GTG GTC AG	
X-box binding protein 1	Xbp1	XBP1 F	NM_005080	GGA GTT AAG ACA GCG CTT GG	248
		XBP1 R		ACT GGG TCC AAG TTG TCC AG	
DNA damage inducible transcript 3	DDIT3	DDIT3 F	NM_004083	CTT TCT CCT TCG GGA CAC TG	203
		DDIT3 R		TGT GAC CTC TGC TGG TTC TG	
DnaJ homologue, subfamily B, member 9	DNAJB9	DNAJB9 F	NM_12328	AAA ATA AGA GCC CGG ATG C	238
		DNAJB9 R		CGC TTC TTG GAT CCA GTG	
Heat shock 70kDa protein 1B	HSPA1B	HSPA1B F	NM_005346	CGA CCT GAA CAA GAG CAT C	213
		HSPA1B R		AAG ATC TGC GTC TGC TTG G	
Insulin-induced gene 1	INSIG1	INSIG1 F	NM_198336	TAC GCT GAT CAC GCA GTT TC	239
		INSIG1 R		TCA CTA TGG GGC TTT TCA GG	
Homocysteine-inducible, ER stress inducible, ubiquitin-like domain	HERPUD1	HERPUD1 F	NM_001010990	GAG CCT GCT GGT TCT AAT CG	194
		HERPUD1 R		GAA AGC TGA AGC CAC CCA TAG	
Mitogen activated protein kinase 10	MAPK10	MAPK10 F	NM_138981	TGA AGA AAT TGC AAC CCA CA	238
		MAPK10 R		GCT GGG TCA TAC CAG ACG TTG	
ER to nucleus signaling 1	ERN1	ERN1 F	NM_001433	CGG CCT TTG CAG ATA GTC TC	226
		ERN1 R		ACG TCC CCA GAT TCA CTG TC	
ER degradation enhancer, mannosidase alpha-like	EDEM1	EDEM1 F	NM_14674	TGG ACT GCA GGT GCT GAT AG	195
		EDEM1 R		GGA TTC TTG GTT GCC TGG TAG	
CCAAT/enhancer binding protein beta	CEBPB	CEBPB F	NM_005194	CTC GCA GGT CAA GAG CAA G	192
		CEBPB R		AGC TGC TCC ACC TTC TTC TG	
